# Chlorophyll a fluorescence as a tool to monitor physiological status in the leaves of *Artemisia ordosica* under root cutting conditions

**DOI:** 10.3389/fpls.2023.1308209

**Published:** 2024-01-15

**Authors:** Ying Liu, Chuangang Gong, Weihao Pei, Kaixuan Fan, Wenjing Shen

**Affiliations:** ^1^ School of Earth and Environment, Anhui University of Science and Technology, Huainan, China; ^2^ The Anhui Province Engineering Laboratory of Water and Soil Resources Comprehensive Utilization and Ecological Protection in High Groundwater Mining Area, Anhui University of Science and Technology, Huainan, China; ^3^ School of Geodesy Geomatics, Anhui University of Science and Technology, Huainan, China

**Keywords:** JIP-test, photosynthesis, plant root damage, energy pipeline models, root soil composite layer

## Abstract

**Background:**

Root cutting caused by underground coal mining subsidence is among the leading causes of plant damage in western China. Detection of root cutting stress is of great importance in evaluating the degree of plant damage and changes in physiological conditions in underground coal mining disturbance conditions.

**Methods:**

The present study assessed the use of chlorophyll fluorescence OJIP transient data to evaluate the disturbance characteristics of root cutting stress on leaf photosynthetic mechanisms in the typical shrub *Artemisia ordosica* Krasch. Different root cutting ratios (10%, 20%, 30%, 50%, 75%, and 100%) were established on the roots of *A. ordosica* in the field, and the OJIP transient and JIP parameters of the leaves were measured.

**Results:**

The overall OJIP curves and each OJIP step in leaves decreased as the root cutting ratio increased, but the impact was relatively small for root cutting ratios of less than 30%. Through the analysis of JIP parameters and the established energy pipeline model, it was found that the energy capture efficiency and electron transfer efficiency of photosystem II decreased as the root cutting ratio increased. Therefore, we also inferred that the threshold for the plant root cutting ratio at which leaf photosynthetic mechanisms begin to change is 30–50%.

**Conclusion:**

These results indicate that OJIP transient analysis can serve as a non-destructive, rapid technique for detecting plant root cutting stress in coal mining subsidence areas, which is of great value for non-destructive monitoring of plant root damage.

## Introduction

The ecological and environmental issues caused by the development of coal resources have become a major area of national scientific and technological research in the semi-arid and arid areas of western China, where the ecological environment is particularly fragile (Liu et al., 2023). One of the most important ecological and environmental issues that western China mining areas are facing is vegetation deterioration (Liu et al., 2021a). The damage to surface vegetation caused by coal mining subsidence in semi-arid mining areas is currently the subject of extensive research (Mi et al., 2021). Researchers have found useful monitoring techniques as well as principles governing the temporal and spatial patterns of surface vegetation disturbance in such subsidence areas (Bian et al., 2020; Liu et al., 2020; Liu et al., 2021a). The damage mechanism of coal mining subsidence to typical plant individuals has been preliminarily clarified, providing a theoretical framework and guidance for future research. The process by which individual damage to plants is induced by coal mining subsidence can be systematically explained from the perspectives of rock breaking and movement above ground (Qian and Xu, 2019), soil settlement and deformation (Lu et al., 2019), changes in plant growth site conditions (Feng et al., 2019), and changes in photosynthetic physiological characteristics of plant leaves (Liu et al., 2020).

The mechanical process of the particular interface between the root system and surrounding soil has been closely linked to the mechanical process of root damage in semi-arid mining areas, as reported by researchers who have recently studied root damage of plants around coal mines on an individual plant scale (Ding et al., 2013; Pan et al., 2017). The mechanism of root damage caused by mining in semi-arid mining areas still merits thorough exploration, particularly the threshold number or percentage of damaged roots per plant that harms an individual and the response mechanism of plant leaf photosynthesis to root damage caused by mining, given the complicated mechanical mechanism of root damage caused by coal mining subsidence, various unclear factors, and even the root system simply going in the soil, which has a “black box effect” (Butnor et al., 2016). For the reasons listed above, it is impossible to fully comprehend the mechanism by which coal mine subsidence leads to plant root damage.

Recent studies have shown that rapid chlorophyll fluorescence (ChlF) can be used as an effective tool to monitor the photosynthetic physiological state of plants under abiotic stress (Kalaji et al., 2016). One of its main advantages is that ChlF technology is non-invasive, which allows scientists to obtain rich information about the photosynthetic process without destroying or even damaging the tested samples (Falcioni et al., 2023). Under natural conditions, plants are affected by many adverse environmental stress factors. These can damage photosynthetic organs, leading to a decrease in plant productivity and thus total yield (Kalaji et al., 2014). Photosynthesis is particularly sensitive to environmental stress, making photosynthesis measurement an important component of plant stress research (Ahmad et al., 2022). However, traditional methods, even technologically advanced methods, such as measuring the photosynthetic rate through gas exchange (CO_2_, H_2_O, and O_2_), require a substantial amount of both time and labor and only provide incomplete information about the overall photosynthetic function (Kalaji et al., 2016). In contrast, ChlF measurement is a simple, non-destructive, inexpensive, and fast tool that can be used to analyze light dependent photosynthetic reactions and indirectly evaluate chlorophyll content in the same sample tissue (Stirbet and Govindjee, 2012). These technological advantages of ChlF make it a popular technology for researchers such as plant physiologists and ecologists. In plant stress research, ChlF measurement also provides critical indirect information about plant physiological conditions (Zushi and Matsuzoe, 2017). By analyzing the ChlF induction curve, the physiological state of photosystem II (PSII) and photosynthetic electron transport chain can both be evaluated (Liu et al., 2020).

We hypothesized that root damage caused by underground coal mine collapse disturbances cause changes in the photosynthetic physiological state of plant leaves, and this change process can be well detected by using ChlF technology. Accordingly, field experiments with manually controlled variables were conducted to test the above hypothesis. As it can be challenging to locate suitable experimental objects with a range of root cutting ratios during field experiments owing to the “black box effect” of the plant root soil system, as stated above, different plant root cutting ratios were therefore implemented under controlled conditions in the present experimental design. The research objectives of this study included the following: (1) assess the viability of using ChlF technology to monitor changes in plant photosynthetic physiological status under root damage caused by disturbances in underground coal mining subsidence; (2) characterize the rapid ChlF dynamics and related biophysical parameters of plant leaves with different root damage ratios; (3) explore the threshold plant root cutting ratio at which leaf photosynthetic mechanisms begin to change.

## Materials and methods

### Study area

The selected research site was located at the 52508 working face of the Daliuta mining region in Shaanxi Province, China (110°05′23″ 110°20′54″ E, 39°15′53″–39°27′32″ N). This region is situated in the transitional zone between the mountainous Loess Plateau region and the Mu Us Desert, on the border of the provinces of Shanxi, Shaanxi, and Inner Mongolia, which receives little rain and has a dry climate. Summer months account for the majority of the 413.5 mm annual average rainfall and 2111.2 mm annual average evaporation. The climate of the site is characteristic of the dry and semi-dry plateau continental climate. The annual average humidity is 56%, the annual average wind speed is 2.3 m/s, and the maximum wind speed is 28.0 m/s. The annual average sunshine hours are 2875.9, the annual average temperature is 7.3°C, the annual extreme maximum temperature is 38.9°C, and the annual extreme minimum temperature is -28.1°C. The vegetation types in the study area mainly included arid grasslands, deciduous broad-leaved shrublands, and vegetation that tolerates sandy soils, mainly represented by *Artemisia ordosica* Krasch., *Caragana korshinskii* Kom., and *Populus simonii* Carr. In recent years, large-scale coal mining in the area has led to surface subsidence and ground fissures, which are known to directly or indirectly damage the roots of surface plants around the mining area. This phenomenon can be seen everywhere on the surface of the working face in the mining area (images of plant roots that have been pulled apart by underground coal mining subsidence observed on the working face surface are provided in **Supplementary Figure 1**).

### Selection of experimental sites and typical plants

To explore the fast ChlF dynamics of plant leaves and the changes in pertinent biophysical parameters under the circumstances of coal mining with various root cutting ratios (RCR), *A. ordosica* was chosen as the research object in this work. All the *A. ordosica* involved in the field experiment were sourced from the surface of the 52307 working face in the Shendong mining area, the largest coal mining base in western China. Field research has revealed that *A. ordosica*, a native plant species with a higher population density than other plants, has a clear quantifiable advantage on the chosen working face surface. In addition, *A. ordosica* is also one of the main plant species for vegetation reconstruction in mining areas in western China. Soil water content (SWC), nutrient content, and particle composition information were measured by sampling the selected plant root soil (**Table 1**).

**Table 1:** Summary of parameters and formulas used for assessing chlorophyll fluorescence transient.

Fluorescence parameters	Description	References
F_0_=F_20μs_	Fluorescence when all PSII RCs are open	(Stirbet and Govindjee, 2012)
F_m_=F_P_	Maximal fluorescence at P-step, when PSII RCs are closed	(Tsimilli-Michael, 2019)
F_v_=F_m_–F_0_	Maximum variable fluorescence	(Tsimilli-Michael, 2019)
F_v_/F_m_=(F_m_–F_0_)/F_m_	Maximal photochemical efficiency of PSII	(Tsimilli-Michael, 2019)
F_v_/F_0_=(F_m_–F_0_)/F_0_	Thermal dissipation quantum yield	(Liu et al., 2020)
V_J_=(F_2ms_–F_0_)/(F_m_–F_0_)	Relative variable fluorescence at J-step (2 ms)	(Kalaji et al., 2016)
M_0_ = 4(F_300μs_–F_0_)/(F_m_–F_0_)	Approximated initial slope of the fluorescent transient	(Zushi and Matsuzoe, 2017)
ABS/RC=M_0_*(1/V_J_)*[1–(F_0_/F_m_)]	Absorption flux per RC	(Zushi and Matsuzoe, 2017)
TR_0_/RC=M_0_*(1/V_J_)	Trapping flux leading to Q_A_ reduction per RC	(Tsimilli-Michael, 2019)
ET_0_/RC=M_0_*(1/V_J_)*(1–V_J_)	Trapping energy used for electron transport per RC	(Tsimilli-Michael, 2019)
DI_0_/CS	Energy dissipation per unit area	(Tsimilli-Michael, 2019)
PI	Performance index based on absorbed light energy	(Liu et al., 2020)
ET_0_/TR_0_	Probability that a trapped exciton moves an electron into the electron transport chain beyond Q_A_^-^ (t=0)	(Strasser et al., 2000)
TR_0_/ABS	Maximum quantum yield for primary photochemistry (t=0)	(Tsimilli-Michael, 2019)
ET_0_/ABS	Quantum yield for electron transport (t=0)	(Tsimilli-Michael, 2019)
Sm	Normalized total complementary area above the curve	(Strasser et al., 2000)
ABS/CS_0_	Absorption of light energy per CS = F_0_	(Strasser et al., 2000)
ET_0_/CS_0_	Electron transport flux per CS at t = F_0_	(Zushi and Matsuzoe, 2017)
TR_0_/CS_0_	Trapping of excitation energy flux per CS at t = F_0_	(Zushi and Matsuzoe, 2017)
DI_0_/CS_0_	Dissipation energy flux per CS at t = F_0_	(Zushi and Matsuzoe, 2017)
RC/CS_m_	Density of RCs per CS at t = F_m_	(Zushi and Matsuzoe, 2017)

### Simulation of plant root cutting as occurs in underground coal mining subsidence disturbance

**Figure 1:**
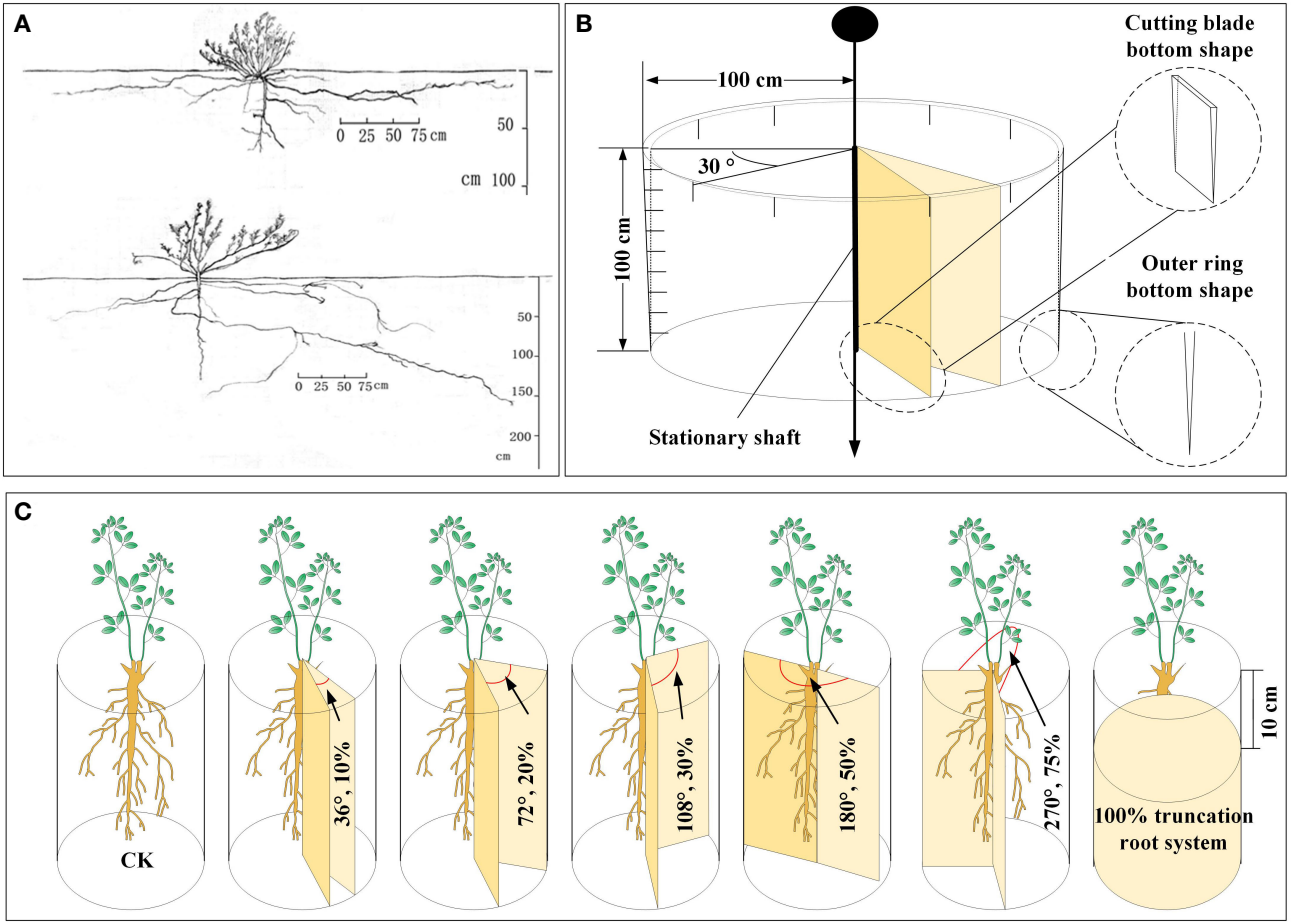
The spatial distribution map of *A. ordosica* root system in the soil profile obtained using the trench method in the early stage of the experiment **(A)**, the schematic diagram of the home-made plant root cutting device **(B)**, and schematic representation of plant root cutting in disturbance caused by underground coal mining subsidence **(C)**.

We conducted a survey on the geographical distribution of the *A. ordosica* root system in the soil profile of the experimental region in the early stages of the present fieldwork. The main root and lateral roots on both sides of *A. ordosica* root systems were uncovered, with average lengths of 1.08 m for the main root and 2.89 m for the lateral roots. The lateral roots’ horizontal distribution range was around 2.15 m, while the main root’s vertical distribution depth was 1.0 m **Figure 1A**. Based on these findings, we designed a device for severing plant roots without disturbing the soil layer outside the cutting zone (the device is shown in **Figure 1B**). The two movable cutting blades in the middle of the device can be rotated to set a specific cutting angle. The outer ring is made of steel, and the bottom of the outer ring and movable cutting blade are both blade shaped, enabling the device to easily cut into the soil profile.

In this root cutting simulation experiment, uniformly growing *A. ordosica* plants were selected, and we set the cutting angles to 36°, 72°, 108°, 180°, and 270°, with corresponding RCRs of 10%, 20%, 30%, 50%, and 75%, respectively (**Figure 1C**). It should be noted that we also set an experimental group with all the roots of *A. ordosica* removed, corresponding to an RCR or 100%, at a depth of 10 cm below the soil surface layer (in order to prevent plant death in the short term as much as possible), and we also used *A. ordosica* without any root cutting treatment as the control (CK). Each treatment group was established with three replicates.

### Chlorophyl fluorescence and JIP test parameters

The OJIP transients of leaves were measured using the second or third fully unfolded mature leaf on the branch (Zushi and Matsuzoe, 2017). All measurements were recorded between 9:00 and 17:00 on July 24 and 31, 2023, respectively, 2 days after the plant root cutting treatment was conducted. In the measurement of OJIP fluorescence transients, the blades to be measured were subjected to a 30-minute dark adaptation treatment before measurement, which includes the attachment of special plastic clips to the leaf blades (Opti-science, Inc., NH, USA). After this treatment, the OJIP transient was measured using a portable chlorophyll a fluorescence meter (OS-30P; Opti science, Inc.). The measured OJIP step size indicated the minimum fluorescence intensity (O step) when all PSII reaction centers (RCs) were open, while the intensity at 2 ms (J step), the intensity at 30 ms (I step), and the maximum intensity (P step) indicated when all PSII-RCs were closed (Zushi and Matsuzoe, 2017). Each selected leaf was measured two times, and the average of the two measurements was recorded as the final measurement value. In addition, JIP parameters based on the JIP test equation were calculated to identify the damaged sites among the PSII electron acceptor sites. JIP parameters and formulas used for assessing the ChlF transient were as follows:

### Statistical analysis

The OJIP transient data and root cutting data were measured daily and at each time period (9:00, 11:00, 13:00, 15:00, and 17:00) during the experiment. All statistical analyses were conducted using the statistical software GraphPad Prism 7.0 (GraphPad Software, San Diego, CA, USA). Analysis of variance (ANOVA) and Dunnett’s test were used to evaluate the differences between the treatment group and the control group at significance levels of 0.05 (*, *P* < 0.05), 0.005 (**, *P* < 0.005), 0.0005 (***, *P* < 0.0005), and 0.0001 (****, *P* < 0.0001).

## Results

### OJIP curves of *A. ordosica* leaves under different RCR

**Figure 2:**
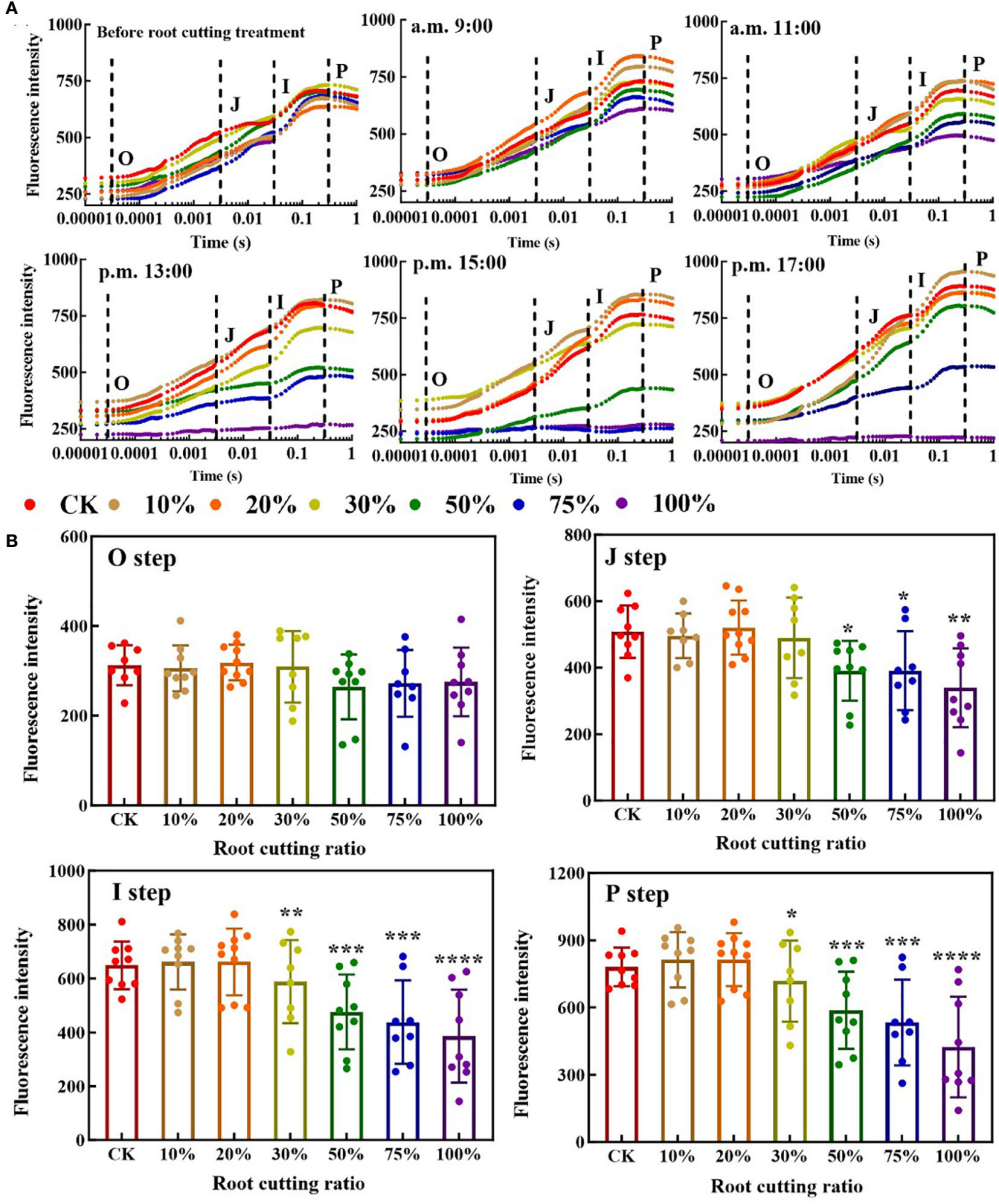
ChlF OJIP transient curves of *A. ordosica* leaves **(A)**; and the O, J, I, and P steps of *A. ordosic*a leaves in different RCR **(B)**. Data are shown as the mean ± standard error (*n* = 9). The differences between the treatment group and the control group at significance levels of 0.05 (*, P < 0.05), 0.005 (**, P < 0.005), 0.0005 (***, P < 0.0005), and 0.0001 (****, P < 0.0001), using the Dunnett’s multiple comparison test.

In the OJIP curves of leaves at different measurement time periods, each step exhibited a different response to each of the different RCRs (**Figure 2A**). At 9:00, there was no significant difference in the O step and J step of the leaf OJIP curve between the six groups with different RCRs and the control, and the change tendency for the I step and P step was lower under 50%, 75% and 100% RCRs relative to the control, but not significantly. At 11:00, the I step and P step at RCRs of 50%, 75%, and 100% became significantly lower than those under the control, and the OJIP curve with 100% RCR began to flatten out. At 13:00, the J step, I step, and P step under RCRs of 50%, 75%, and 100% were significantly lower than those under the control, and the OJIP curves with 50% and 75% RCR began to flatten out; meanwhile, under 100% RCR, the OJIP curve became almost horizontal. At 15:00, the OJIP curve with 50% RCR was further flattened, while those with 75% and 100% RCRs became and remained horizontal, respectively. At 17:00, the OJIP curves with 50% and 75% RCRs showed a “self-recovery” phenomenon, and there was no significant difference between the OJIP curves with 50% RCR and the control; at the same time, the OJIP curves under 75% RCR showed a significant difference from the control, but, in contrast, the OJIP curves with 100% RCR remained horizontal and did not exhibit such self-recovery (**Figure 2A**). The average values of O, J, I, and P steps of *A. ordosica* leaves under different RCRs during each measurement time are shown in **Figure 2B**. When the RCR was greater than 30%, the tendency of fluorescence intensity values in the J step, I step, and P step became significantly lower, but when the RCR was between 0% and 20%, there was almost no difference in fluorescence intensity values for each of the four steps.

### JIP parameters and energy pipeline models of *A. ordosica* leaves under different RCR

**Figure 3:**
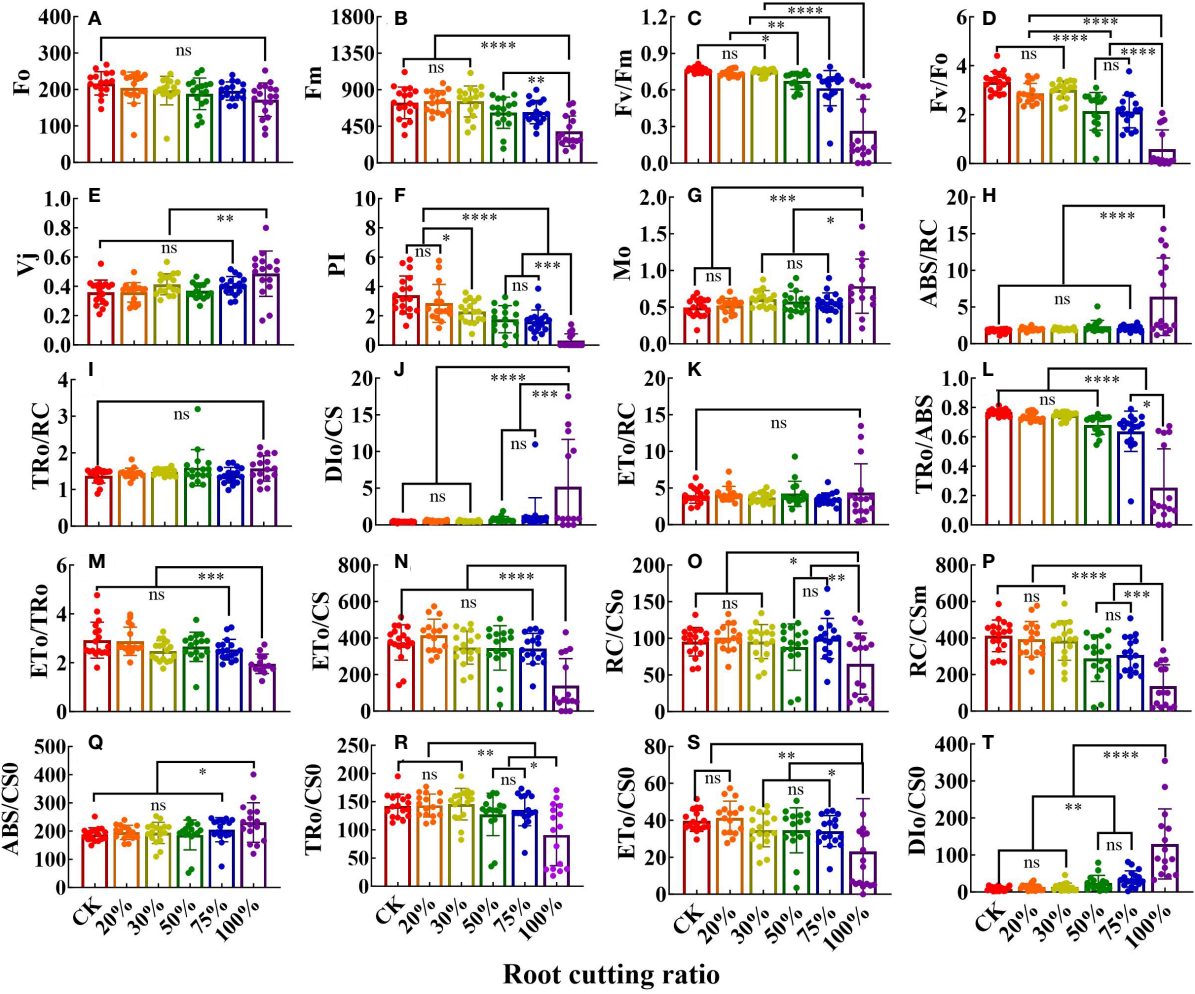
The JIP parameters deduced from ChlF OJIP transient curves in the leaves grown under different RCR (*n* = 18). F_0_ **(A)**; F_m_ **(B)**; F_v_/F_m_ **(C)**; F_v_/F_0_ **(D)**; V_j_ **(E)**; PI **(F)**; Mo **(G)**; ABS/RC **(H)**; TR_0_/RC **(I)**; DI_0_/CS **(J)**; ET_0_/RC **(K)**; TR_0_/ABS **(L)**; ET_0_/TR_0_ **(M)**; ET_0_/CS **(N)**; RC/CS_0_ **(O)**; RC/CS_m_ **(P)**; ABS/CS_0_ **(Q)**; TR_0_/CS_0_ **(R)**; ET_0_/CS_0_ **(S)**; DI_0_/CS_0_ **(T)**. The differences between the treatment group and the control group at significance levels of 0.05 (*, P < 0.05), 0.005 (**, P < 0.005), 0.0005 (***, P < 0.0005), and 0.0001 (****, P < 0.0001), using the Dunnett’s multiple comparison test. “ns” represents no significant difference between the control group and the treatment group.

The JIP test parameter values (compared with multiple sets of results) are shown in the bar chart in **Figure 3**. Different RCRs had different effects on JIP parameter values, and specific changes in the tissues were observed. For example, the values of 16 parameters in leaves were significantly affected by root cutting (**Figures 3A–P**). In JIP parameters under 50%, 75%, and 100% RCRs, the values of F_m_, F_v_/F_m_, F_v_/F_0_, performance index (PI), TR_0_/ABS, RC/CS_0_, and RC/CS_m_ were significantly lower than those in the control, whereas the values of M_0_ and DI_0_/CS were higher than those in the control. In JIP parameters under 100% RCR, the values of V_j_ and ABS/RC were higher than those of other experimental groups, whereas the values of ET_0_/TR_0_ and ET_0_/CS were significantly lower than those in other experimental groups. However, there was no significant difference in the values of TR_0_/RC and ET_0_/RC of JIP parameters among the six root cutting treatment groups. In JIP parameters under 20% and 30% RCRs, all parameter values except those of PI and M_0_ were not significantly different than those of the control.

**Figure 4:**
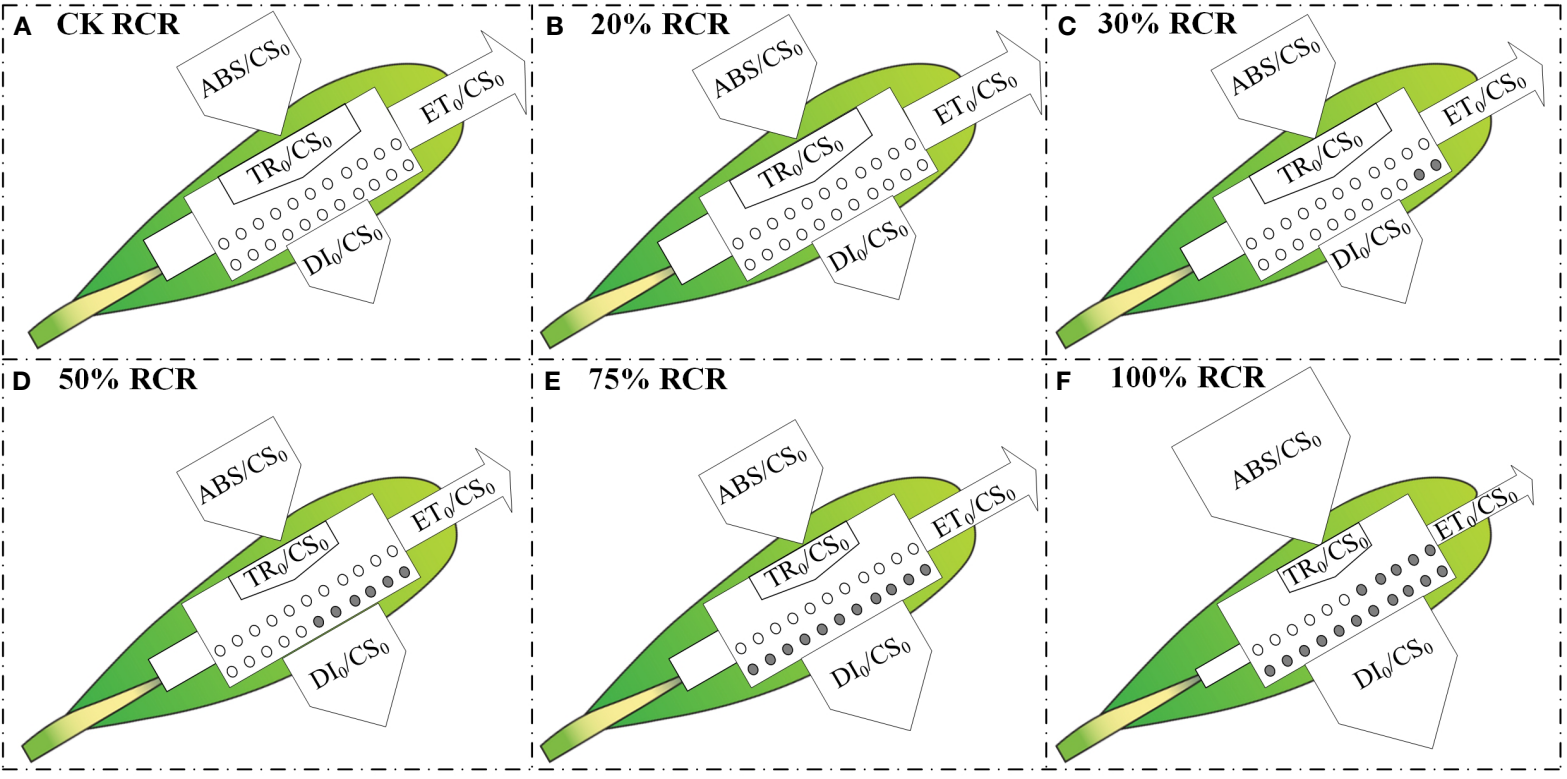
Energy pipeline models of phenomenological energy fluxes per excited cross-section (CS_0_= O step) in the *A. ordosica* leaves grown under different RCR. CK RCR **(A)**; 20% RCR **(B)**; 30% RCR **(C)**; 50% RCR **(D)**; 75% RCR **(E)**; 100% RCR **(F)**. ABS, absorption; TR, maximum trapping flux beyond Q_A_; ET, electron transport; and DI, electron dissipation. In the models, active and inactive reaction centers (RCs) are indicated by open and filled circles, respectively.

In the next step, to visualize the symptoms of root cutting stress and the energy flux based on the OJIP curve, such as light absorption, light capture, electron transport, and dissipation of each excitation cross-section (CS_0_ = F_0_), energy pipeline models for root cutting stress leaves were developed (**Figures 4A–F**). In the leaves, the absorption of light energy per CS = F_0_ (ABS/CS_0_), except that under 100% RCR, did not change significantly with increases in RCR from CK to 75% RCR; at the same time, the electron transport flux per CS at t = F_0_ (ET_0_/CS_0_) and the trapping of excitation energy flux (TR_0_/CS_0_) decreased as RCR increased, and the dissipation energy flux per CS at t = F_0_ (DI_0_/CS_0_) increased with RCR, as indicated in **Figures 3Q–T**.

## Discussion

### Root cutting induced changes in OJIP curve of *A. ordosica* leaves

Rapid ChlF can be an effective tool for monitoring the photosynthetic physiological status of plants under various environmental stresses, such as salt stress (Zushi and Matsuzoe, 2017), drought stress (Erdal and Ekmekçi, 2023), and heavy metal stress (Gan et al., 2023), among others, in several plant species. OJIP transient detection technology, as a non-destructive, simple, and fast detection method, is widely used to evaluate the degree of damage to photosynthetic organs under various environmental stresses (Kalaji et al., 2016). However, there are few studies using this technology to monitor the degree of damage induced by root cutting as manifest in photosynthetic organs or plant leaves. Owing to the underground growth of plant roots, it is usually only possible to accurately evaluate plant root breakage caused by underground coal mining by excavating the entire plant from the soil. In the arid mining areas of western China, vegetation is already scarce, and the ecological environment is particularly fragile (Liu et al., 2021b). Obviously, the above method for determining plant root breakage by excavation would directly damage plants, which is clearly not advisable for plant protection and restoration in such a region. Therefore, it is necessary to explore non-destructive methods for monitoring plant root tensile damage. The OJIP transient detection technology was first used to monitor changes in the photosynthetic mechanism of plant leaves induced by manual root cutting, intended to simulate the severing of roots by subsidence of local coal mines. The monitoring results showed that the OJIP curve of plant leaves showed significant changes under different RCRs, especially when the RCR was greater than 30% (**Figure 2A**). Thus, it was found that this non-destructive, simple, and fast detection method is an effective method for monitoring the shear state of plant roots, especially when the RCR is greater than 30% (**Figures 2A, B**). In other words, from the experimental results, when the RCR is less than 30%, the monitoring effect of this method is not particularly obvious.

In each OJIP step, the monitoring results indicated that the O, J, I, and P steps at 50%, 75%, and 100% RCR were lower than those in the control, but the difference in fluorescence intensity values at the O step was not significant (**Figure 2B**). These results indicate that the J, I, and P steps in leaves were more sensitive parameters than the O step in response to root cutting. The O step indicates the fluorescence from excited chlorophyll molecules in light-harvesting complex II (LHCII) that has neither resulted in excitation of P680 nor been converted to heat (Strasser et al., 2004; Zushi and Matsuzoe, 2017). The O–J step of the fluorescence rise is related to the closure of some of the PSII reaction centers in response to the reduction of Q_A_ to a level determined by the ratio between the trapping rate and Q_A_ reoxidation rate by Q_B_ and the rest of the electron transfer chain (Stirbet et al., 2014; Kalaji et al., 2016). The J–I step of the curve corresponds to the reduction of the secondary electron acceptor Q_B_, plastoquinone (PQ), cytochrome, and plastocyanin (PC) (Suárez et al., 2022). The increase in fluorescence in the I–P step of the induction curve is typically attributed to the reduction of electron transporters (ferredoxin, intermediary acceptors, and NADP) of the photosystem I (PSI) acceptor side (Bhagooli et al., 2021). Therefore, these results suggest that the chlorophyll molecules excited in LHCII are relatively less damaged during root cutting and that the secondary electron acceptor Q_B_, PQ, cytochrome, PC, and the electron transporters (ferredoxin, intermediary acceptors, and NADP) of the PSI acceptor side were damaged by severe root cutting. Consequently, the values of J, I, and P steps were lower under severe root cutting.

### Root cutting induced changes in JIP parameter values and energy pipeline models of *A. ordosica* leaves

JIP parameters can be used to assess sensitive biochemical functions such as energy absorption, energy trapping, and electron transport in PSII and PSI (Zushi and Matsuzoe, 2017); therefore, the JIP-test approach has been used in various areas of plant biology to understand the responses of the photosynthetic apparatus to different environmental conditions (Strasser et al., 2000; Strasser et al., 2004). The changes in the PSII reaction center indicate that as the root interception rate of *A. ordosica* increases, the light energy absorbed per unit reaction center (ABS/RC), captured light energy (TR_0_/RC), and heat dissipated energy (DI_0_/CS) all increased, while the energy transferred by electrons (ET_0_/RC) actually decreased (**Figure 3**). This indicates that root cutting stress affected plant leaf PSII and increased light energy absorption, but the transmission of photosynthetic electrons was hindered, leading to the accumulation of excess light energy in the leaves. By increasing heat dissipation, the pressure caused by excess light energy was alleviated. This is a response mechanism of *A. ordosica* to root cutting stress and also a self-regulation mechanism shaped by domestication and adaptation under long-term stress (Luo et al., 2016). As the RCR of *A. ordosica* increased, the probability of electron transfer from *A. ordosica* leaves to electron acceptors downstream of Q_A_^-^ in the electron transfer chain (ET_0_/TR_0_), quantum yield for electron transfer (ET_0_/ABS), and maximum quantum efficiency of PSII (TR_0_/ABS) all significantly decreased, further indicating that photosynthetic electron transfer was hindered, leading to the accumulation of excess light energy. The initial slope (Mo) of the OJIP curve reflects the reduced rate of the primary quinone receptor Q_A_ during the O–J step (Strasserf and Srivastava, 1994). In to present study, as RCR increased, Mo significantly increased, indicating that the Q_A_ reduction rate accelerated Q_A_^-^ accumulation and that electron transfer was inhibited.

In addition, the maximum photochemical efficiency (F_v_/F_m_) under normal conditions was greater than 0.8 after dark adaptation. However, when F_v_/F_m_ values are continuously less than 0.8, plants are under environmental stress, leading to photoinhibition (Bj Rkman, 1987; Johnson et al., 1993). In the present study, when the RCR was between 10% and 30%, F_v_/F_m_ was greater than or close to 0.8. As the RCR rose to greater than 30%, F_v_/F_m_ decreased subsequently and was less than 0.8. This indicated that under root cutting stress, the leaves of *A. ordosica* are subjected to stress, leading to photoinhibition. This also indicated that F_v_/F_m_ can be used as an effective indicator to monitor and evaluate the stress status of plants, as it was utilized in this study. The performance index (PI) is an indicator based on light absorption that accurately reflects the overall condition of plant photosynthetic organs (Christen et al., 2007), and Oukarroum et al. (2007) posited that PI is much more sensitive to stress than is F_v_/F_m_. Therefore, the present study conducted statistical analysis on the PI values of *A. ordosica* leaves, and the PI was found to significantly decrease when the RCR was greater than 30%. As the RCR increased, the PI of *A. ordosica* leaves decreased and was inhibited by light, hindering the growth of *A. ordosica*. In addition, we also found that significant changes in both OJIP transient curves and JIP parameters (such as F_m_, F_v_/F_m_, F_v_/F_o_, Mo, PI, TR_0_/ABS, RC/CS_0_, RC/CS_m_, DI_0_/CS, ET_0_/CS, etc.) occurred when the RCR was greater than or equal to 30%. However, does this mean that the threshold of root cutting damage causing changes in the photosynthetic mechanism of *A. ordosica* leaves is greater than 30% and that the specific threshold of RCR is at some optimum between 30% and 50%? Future experiments should explore this findings through control experiments evaluating RCR values from 30% to 50%.

The above analysis shows that when a large number of cracks are formed on the surface during coal mining, the soil structure is damaged, leading to tension on plant roots in the area of the crack. Under these conditions, the growth of *A. ordosica* would be expected to be under stress, affecting the normal process of photosynthesis. According to the theory of mining subsidence (Qian and Xu, 2019), underground coal mining disrupts the equilibrium state of stress in the overlying rock layers, leading to rock fracture and movement, redistributing stress among the soil layers above (including plant root soil composite layers). Once this stress change exceeds a certain threshold, the root system will be pulled apart, and the direct consequence is that the channels for plant tissue to obtain nutrients and soil water are in turn disrupted (Pan et al., 2017). Especially for the arid and semi-arid areas in western China, soil moisture is undoubtedly the most important factor limiting plant growth (Liu et al., 2020). The disruption of water transport channels caused by tension on roots can further decrease the amount of water plants can obtain. Thus, the higher the RCR for plant roots, the less water a plant will obtain. Plant growth is constrained by drought stress if sufficient water cannot be obtained to maintain normal physiological functions. In addition, we also found that the changes in OJIP curves and JIP parameters obtained in this study under different RCRs were similar to the changes in the above indicators under drought stress (Liu et al., 2020). Therefore, we infer that the main cause of the changes in plant photosynthetic physiology under root cutting disturbance was the disruption of water transport channels caused by root severing, which further led to drought stress on plant growth. Thus, JIP-test technology was able to effectively identify the impact of root severing on the ChlF response of *A. ordosica* leaves, and F_v_/F_m_ and PI can both serve as effective indicators of light inhibition in *A. ordosica* leaves.

The energy pipeline model indicates that several elements of PSII are sensitive to various types of environmental stress (Zushi et al., 2012; Gautam et al., 2014; Zushi and Matsuzoe, 2017). In the present study, the energy pipeline model was used to assay the active and inactive sites in the phenomenological flux of each CS_0_ (ABS/CS_0_, TR/CS_0_, DI_0_/CS_0_, and ET_0_/CS_0_). The research results indicate that as the RCR of *A. ordosica* increased, the TR_0_/CS_0_ and ET_0_/CS_0_ of the leaves decreased (**Figure 3**, **4**), as the active reaction center (RC) was converted into an inactive RC, thereby reducing the energy capture efficiency and electron transport of PSII. Similarly, in *A. ordosica* leaves, as the RC increased, there was a decreased in energy capture and electron transfer (F_m_) per excitation cross-section, indicating a decrease in energy absorption efficiency of PSII (**Figure 3**).

## Limitations

**Figure 5:**
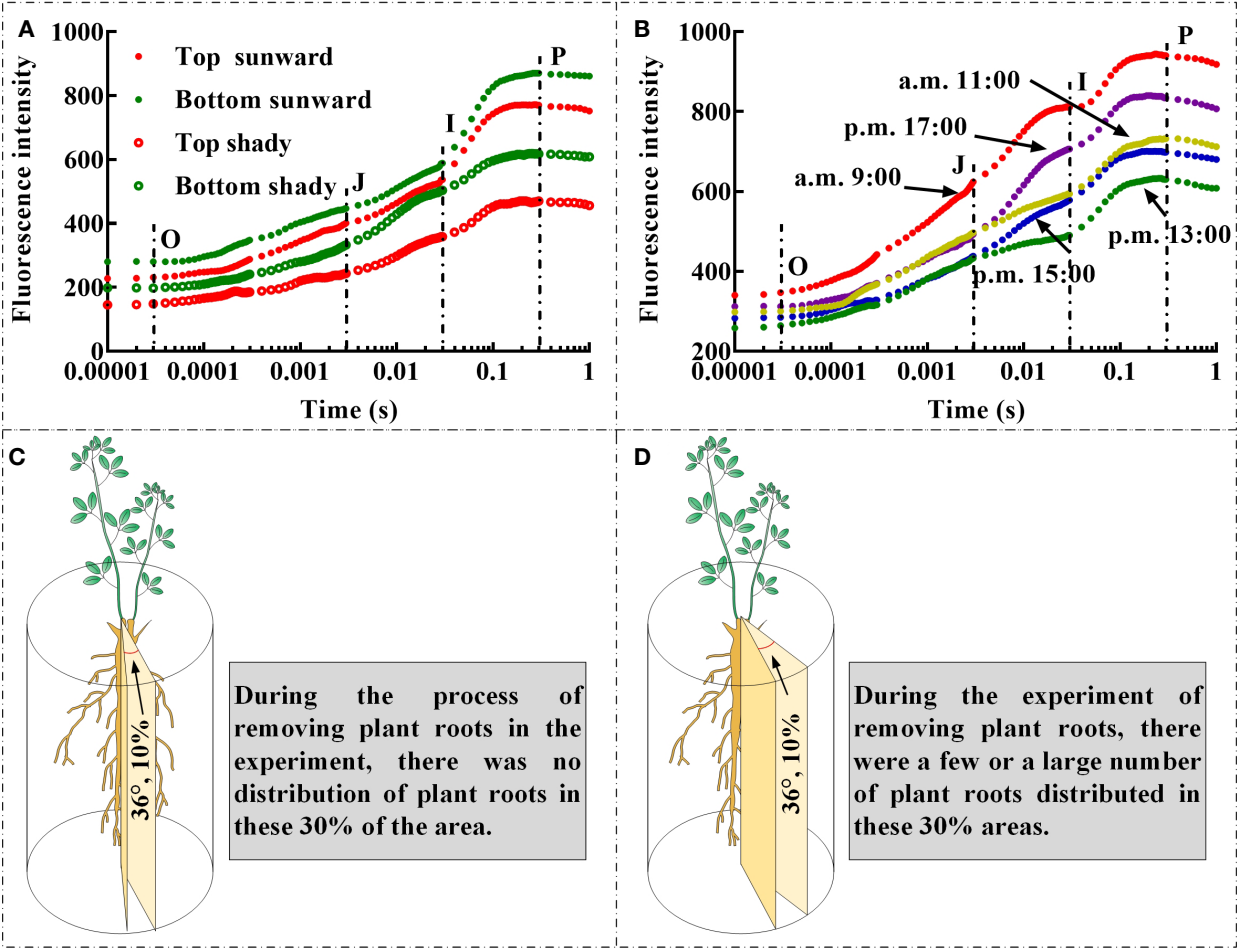
The influence of leaf position **(A)**, whether the leaves are exposed to sunlight **(A)**, and light intensity on the OJIP curve **(B)**; During the experimental process of cutting off plant roots, the spatial location of root cutting with different RCR may lead to differences between the actual root cutting rate and the set root cutting rate, the cutting area has no root distribution **(C)**, while the cutting area has a few or large amount of root distribution **(D)**.

The rapid ChlF test was developed specifically as a biophysical tool for evaluating the cascade of chloroplast redox reactions at microsecond or millisecond scales (Kalaji et al., 2016; Esmaeilizadeh et al., 2021; Sharma et al., 2021; Shamsabad et al., 2022a; Sieczko et al., 2022; Dąbrowski et al., 2023). The link between the physiological condition of samples and the fluorescence transient has also been the subject of important empirical research in the past, including demonstrating the direct or indirect association between the physiological state of leaves and ChlF transients. (Strasser et al., 2000; Bhagooli et al., 2021; Falcioni et al., 2023). The interplay of the structure and function of photosynthetic components based on present metabolic requirements or environmental variables results in the observed OJIP curves, JIP parameters, and other signals (Kalaji et al., 2016). Therefore, several factors need to be carefully monitored to avoid errors and excessively simplified explanations (Evans, 2009). First, there are differences in the OJIP curves and JIP parameters measured at different positions on the selected branch of the same plant (Pongprasert and Srilaong, 2007); the leaves at the top of the branch are relatively young, whilst the leaves at the bottom of the same branch are rather mature. Second, there is notable variation in light intensity at various times of the same day, and whether the leaves were directly exposed to sunlight during the measurement can also affect the monitoring findings (Liu et al., 2022). We discovered that the OJIP curves of leaves that are directly exposed to sunlight and placed at the bottom of the branches had greater O step, J step, I step, and P step values (**Figure 5A**). In addition, the O step, J step, I step, and P step values of the OJIP curves of the leaves on the same day were higher at approximately 9:00 and 17:00 (**Figure 5B**). Owing to the summer season in China during which we conducted the present field monitoring, light intensity was substantially increased at times closer to noon, which resulted in lower O step, J step, I step, and P step values of the leaf OJIP curve. This may have been related to the closure of leaf stomata caused by excessive light intensity (Luo et al., 2016), high temperature (Shamsabad et al., 2022b), and/or increased evaporative of water from leaves (Liu et al., 2020). Therefore, in the process of field experiments, leaf selection should strive to select leaves that are under the same lighting conditions and that the degree of leaf development is as consistent as possible. Moreover, it should be noted that during the simulation of underground coal mining in the field, owing to the “black box effect” of the root soil system, there may be a situation in which the selected cutting area happens to have no or very few roots (**Figure 5C**). In such a situation, cutting may result in no roots being severed. At the same time, there may also have been situations in which there was a substantial distribution of a root system affected by the cutting area, and the cutting treatment may have resulted in a significant portion of the root system or all roots being severed (**Figure 5D**). As noted in the experimental plan design, the negative consequences of this situation can be avoided through adequate replication.

## Conclusions

Analyzing and revealing the disturbance of plant root damage caused by coal mining collapse on the photosynthetic mechanism of plant leaves is crucial for understanding and addressing the mechanism of individual plant damage caused by underground coal mining. Here, a first attempt was made to use ChlF OJIP transient data to evaluate the interference characteristics of root cutting stress on the photosynthetic mechanism of the typical shrub *A*. *ordosica* leaves, and the feasibility of the application of this technology was verified. The OJIP curve and each OJIP step in the leaves significantly decreased as the root cutting rate increased continuously beyond 30%. Through the analysis of JIP parameters and the established energy pipeline model, it was found that the energy capture efficiency and electron transfer efficiency of PSII decreased as the root cutting rate increased. Thus, the rapid ChlF test can be used as a non-destructive detection method for evaluating plant root damage in underground coal mining subsidence areas.

## Data availability statement

The field sampling data used in this study is available from the corresponding author on reasonable request.

## Author contributions

LY: Conceptualization, Data curation, Funding acquisition, Writing – original draft, Writing – review & editing. GCG: Investigation, Methodology, Writing – original draft. PWH: Investigation, Software, Validation, Writing – original draft. FKX: Formal Analysis, Investigation, Resources, Visualization, Writing – original draft. SWJ: Data curation, Formal Analysis, Investigation, Writing – original draft.
