# Supplementary material for: Chlorophyll a fluorescence as a tool to monitor physiological status in the leaves of Artemisia ordosica under root cutting conditions
Source: Front Plant Sci. 2024 Jan 15;14:1308209. doi: 10.3389/fpls.2023.1308209 (PMC10824239; doi:10.3389/fpls.2023.1308209)
Supplement: Supplementary file 1 [file DataSheet_1.docx]

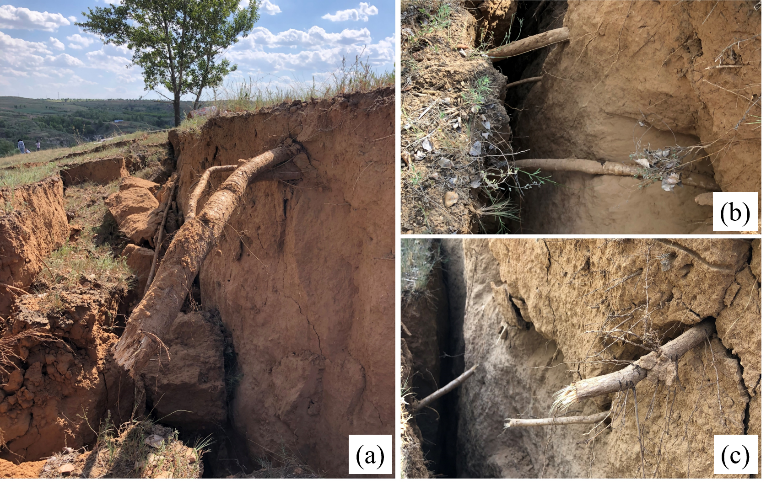


Attached Fig. 1. The characteristics of plant root damage under subsidence disturbance observed on the surface of field working faces

Attached Table 1. The soil environment factors for the selecting plant roots

| Factors | OM  g kg^–1^ | TN  g kg^–1^ | TP  g kg^–1^ | NN  mg kg^–1^ | SWC  mg kg ^–1^ | C:N | C:P | BD  g cm^–3^ | Sand  % | Silt and clay  % |
| --- | --- | --- | --- | --- | --- | --- | --- | --- | --- | --- |
| CK | 3.20±0.39 | 0.29±0.03 | 0.20±0.03 | 0.23±0.04 | 12.28±0.03 | 10.72±0.29 | 16.37±1.69 | 1.64±0.41 | 73.54 | 26.24 |
| RCR10% | 3.58±0.12 | 0.32±0.02 | 0.19±0.02 | 1.56±0.34 | 13.15±0.15 | 11.25±0.95 | 19.20±2.85 | 1.59±0.42 | 75.34 | 24.66 |
| RCR20% | 3.93±0.44 | 0.34±0.04 | 0.20±0.02 | 2.97±1.57 | 12.04±0.04 | 11.31±0.24 | 25.11±1.19 | 1.66±0.27 | 72.91 | 27.09 |
| RCR30% | 3.57±0.22 | 0.32±0.02 | 0.20±0.03 | 1.59±0.31 | 14.05±0.06 | 11.10±0.45 | 20.22±2.08 | 1.63±0.29 | 76.88 | 23.12 |
| RCR50% | 2.49±0.14 | 0.22±0.01 | 0.21±0.04 | 2.81±0.55 | 12.94±0.02 | 11.45±0.10 | 12.43±1.69 | 1.62±0.21 | 71.53 | 28.47 |
| RCR75% | 2.44±0.14 | 0.22±0.02 | 0.19±0.02 | 1.19±0.49 | 14.95±0.09 | 11.25±0.13 | 19.20±1.91 | 1.60±0.38 | 74.66 | 25.34 |
| RCR100% | 4.96±1.02 | 0.49±0.12 | 0.23±0.04 | 1.72±0.26 | 13.03±0.01 | 10.48±1.36 | 22.29±4.58 | 1.64±0.24 | 72.39 | 27.61 |

OM, Organic Matter; TN, Total Nitrogen; TP, Total Phosphorus; NN, Nitrate nitrogen; SWC, Soil Water Conteng; C:N, Total Carbon:Total Nitrogen; C:P, Total Carbon:Total Phosphorus; BD, Bulk Density. Data are mean ± S.D. (*n* = 3).
